# Multiple behavioural impulsivity tasks predict prospective alcohol involvement in adolescents

**DOI:** 10.1111/add.12283

**Published:** 2013-08-14

**Authors:** Gordon Fernie, Margot Peeters, Matthew J Gullo, Paul Christiansen, Jon C Cole, Harry Sumnall, Matt Field

**Affiliations:** 1 Department of Psychological Sciences, University of LiverpoolLiverpool, UK; 2 School of Psychology, University of UtrechtUtrecht, the Netherlands; 3 Centre for Public Health, Liverpool John Moores UniversityLiverpool, UK; * Division of Applied Medicine (Psychiatry), School of Medicine and Dentistry, University of AberdeenUK; † Centre for Youth Substance Abuse Research, The University of QueenslandAustralia

**Keywords:** Adolescents, alcohol, delay discounting, disinhibition, impulsivity, risk-taking

## Abstract

**Aims:**

We investigated reciprocal prospective relationships between multiple behavioural impulsivity tasks (assessing delay discounting, risk-taking and disinhibition) and alcohol involvement (consumption, drunkenness and problems) among adolescents. We hypothesized that performance on the tasks would predict subsequent alcohol involvement, and that alcohol involvement would lead to increases in behavioural impulsivity over time.

**Design:**

Cross-lagged prospective design in which impulsivity and alcohol involvement were assessed five times over 2 years (once every 6 months, on average).

**Setting:**

Classrooms in secondary schools in North West England.

**Participants:**

Two hundred and eighty-seven adolescents (51.2% male) who were aged 12 or 13 years at study enrolment.

**Measurements:**

Participants reported their alcohol involvement and completed computerized tasks of disinhibition, delay discounting and risk-taking at each assessment. Cross-sectional and prospective relationships between the variables of interest were investigated using cross-lagged analyses.

**Findings:**

All behavioural impulsivity tasks predicted a composite index of alcohol involvement 6 months later (all *P*s < 0.01), and these prospective relationships were reliable across the majority of time-points. Importantly, we did not observe the converse relationship across time: alcohol involvement did not predict performance on behavioural impulsivity tasks at any subsequent time point.

**Conclusions:**

Several measures of impulsivity predict escalation in alcohol involvement in young adolescents, but alcohol use does not appear to alter impulsivity.

## Introduction

There are several distinct facets of behavioural impulsivity, many of which overlap with subcomponents of executive (dys)function 1. For example, impulsive decision-making can be assessed with delay discounting procedures, in which participants make choices between small rewards that are available immediately versus larger rewards that are available after a delay 2. Disinhibition is assessed with computerized tasks such as the Stop-Signal 3 and Go/No-Go tasks, both of which establish a dominant motor response that participants are required to occasionally inhibit. Risk-taking can be assessed with tasks such as the Balloon Analogue Risk Task (BART) 4, in which participants attempt to win rewards by risking what they have accumulated up to that point. Dependent measures obtained from these tasks reflect distinct underlying concepts 5, which suggests that impulsivity is not a unitary construct 6. None the less, performance on each of these measures is associated with heavy drinking and alcoholism 7–9 and with other substance use disorders 10.

Initial experimentation with alcohol begins during adolescence. For example, in the United Kingdom approximately 24% of 12-year-olds report at least one episode of alcohol consumption, rising to 77% of 15-year-olds 11. Individual differences in behavioural impulsivity are associated with drinking behaviour and alcohol problems in adolescents 12. Theoretically, elevated impulsivity may pre-date alcohol involvement and serve as a risk factor for the development of heavy drinking and alcohol problems once individuals begin to experiment with alcohol 13. Consistent with this, longitudinal studies demonstrate that high levels of disinhibition are predictive of the development of heavy drinking and alcohol problems several years later 14–16. Regarding other behavioural impulsivity measures, individual differences in the rate of increase in risk-taking during early adolescence (but not absolute levels of risk-taking) are predictive of subsequent alcohol involvement 17. Furthermore, individual differences in delay discounting predict the likelihood of starting smoking 18, although the relationship between delay discounting and subsequent heavy drinking in adolescents has not been investigated.

Adolescence is a critical stage of brain development, and the maturational changes that occur may render adolescents particularly sensitive to neuroadaptations that underlie development of alcohol dependence. For example, developmental brain changes that influence reward processing and impulse control are essential to the long-term development of self-regulation and adaptive decision-making 19,20. Neuroimaging studies have shown that adolescents, relative to adults and young children, show a heightened neural response to rewards in the nucleus accumbens 21,22. This heightened sensitivity occurs within the context of immature processing of reward and risk within the orbitofrontal cortex, a key region involved in inhibitory control 22,23. These features of brain development may render adolescents vulnerable to increased disinhibition, impulsive decision-making and risk-taking as consequences of heavy drinking. Consistent with this, studies with rodents demonstrate that the extent of neuronal loss following binge alcohol exposure is more pronounced in adolescents than in adults 24, as is increased probability discounting caused by heavy drinking 25. There is also some preliminary evidence for neurocognitive deficits arising from alcohol exposure in human adolescents 26,27. However, there is no direct evidence that heavy drinking during adolescence leads to increased impulsivity.

Our goal in the present study was to investigate the relationships between alcohol involvement and performance on behavioural impulsivity tasks among adolescents. We performed a cross-lagged prospective study involving a large sample of adolescents who were aged 12 or 13 years at the beginning of the study and tested each participant five times over 2 years (every 6 months). We recruited participants in this age range as UK government data 11 indicate that alcohol consumption in British adolescents tends to increase rapidly between the ages of 12 and 15 years. We hypothesized reciprocal prospective relationships between alcohol involvement and impulsivity. Specifically, we predicted that (i) individual differences in impulsivity would predict alcohol involvement at subsequent time-points, and (ii) individual differences in alcohol involvement would predict impulsivity at subsequent time-points.

## Method

### Participants (see Supporting information for additional information)

Two hundred and eighty-seven participants (51.2% male) were recruited initially from a total of five mixed-sex secondary schools across the Merseyside region of North West England. Each school allowed us to recruit pupils from year 9, in which all pupils were aged either 12 or 13 years [mean age at enrolment was 13.33 years; standard deviation (SD) = 0.33]. All participants provided informed assent, and the parents or guardians of each participant provided either opt-in or opt-out consent depending on the preference of the individual school. The University of Liverpool Research Ethics Committee approved the study.

### Self-report measures

#### Alcohol use questionnaire

This questionnaire measured the frequency and quantity of alcohol consumption. Participants were first asked whether they had ever had a proper alcoholic drink (‘a whole drink, not just a sip’ 11). If they answered ‘yes’, participants indicated how often they had consumed alcohol over the previous 6 months, using a question taken from the Adolescent Alcohol Involvement Scale 28, and they also estimated the number of times they ‘got drunk’ during this period. Participants also completed a retrospective diary in which they recorded all alcoholic beverages consumed over the previous 2 weeks.

#### Alcohol Problems Index (API) 29

Participants indicated if any of six possible adverse consequences of drinking had happened to them in the previous 6 months (e.g. ‘I lost money or other items’). All affirmative responses are scored 1, therefore the range of possible scores is 0–6.

#### Demographics and socio-economic status (SES)

Participants indicated their sex and date of birth before completing the three-item Family Affluence Scale 30, a well-validated measure of socio-economic status 31. Scores ranged from 0 to 6 (higher scores indicate higher SES). The mean value in our sample was 3.89 (SD = 1.46).

### Behavioural measures (full descriptions can be found in Supporting information)

#### Delay discounting task (DD) 32

This version of the task has been used with adolescent substance abusers to predict treatment outcome 33. We simplified the interface and reduced the amounts of hypothetical rewards that were on offer. The resulting task was similar to one administered to children (mean age of 12 years) with attention deficit hyperactive disorder (ADHD) and a healthy control group 34. In this task, participants made hypothetical choices between a relatively small sum of money that was available immediately versus a fixed larger sum (£50) which was available after a delay. The value of the immediate reward and the length of the delay were adjusted in successive trials. Delay discounting was calculated using area under the curve (AUC) 35.

#### BART 4

This task was validated initially in adults 4, although a modified version where participants win points rather than money has been validated in children with a mean age of 15 years 36. The version used in the present study was identical to that used in a previous study, which revealed that risk-tasking was associated cross-sectionally with alcohol use in college students 8. On each trial of the task, participants used the mouse to inflate an on-screen balloon in order to add hypothetical money to a temporary bank. Participants were instructed that at some point the balloon would burst, and if this happened all the money in the temporary bank would be lost. Participants could collect from the temporary bank at any point before the balloon burst by clicking on a button marked ‘Collect’. The adjusted number of pumps (average number of pumps on trials when the participant banked their temporary funds before the balloon burst) provides the measure of risk-taking.

#### Stop-Signal task 37

This version of the task was identical to one used in a previous study 37 in which participants were children with ADHD and healthy controls, with a mean age of 12 years (range 6–17). In the task, participants manually categorized visual ‘Go’ stimuli as quickly as possible. In 25% of the trials, an auditory ‘Stop’ tone was presented; this tone signalled that participants should refrain from responding. The dependent variable was Stop-Signal reaction time (SSRT) 38. High SSRTs indicate high levels of disinhibition.

### Procedure

Participants completed five identical testing sessions, each lasting no more than 1 hour, spread over a 2-year period. We aimed for a 6-month interval between testing sessions. In 96% of cases the interval between sessions was between 4 and 7 months, although in a minority of instances the delay was shorter (but no less than 3 months) or longer (but no more than 9 months). All participants were tested at their school, in groups ranging in size between four and eight. On rare occasions, participants were absent from school on the date of the original planned testing session; in these cases the researcher returned at a later date and the participant was tested individually. All participants provided written informed consent before enrolling in the study and at the beginning of each individual testing session.

Participants sat at individual desks facing a laptop computer. We arranged the seating to minimize interaction between participants and to ensure that individuals could not see the laptop screens of others. Participants initially completed the self-report measures, before a battery of computerized tasks in one of five predetermined counterbalanced sequences. In addition to the three tasks described here, participants also completed a visual probe task and a stimulus–response compatibility task, results from which are not presented here due to poor internal reliability and between-session stability (see Supporting information for details). At the initial testing session, the experimenter demonstrated how to complete all self-report measures and computerized tasks. In subsequent sessions these instructions were briefly reiterated, with more detailed explanations or demonstrations if necessary. Participants wore headphones during all tasks. Experimenters were present throughout the experimental sessions in order to ensure that participants were focused on the tasks.

At the end of each session, participants received a £5 voucher that they could spend in a national chain of music stores. Participants also received an additional £25 voucher at the end of the final (fifth) testing session if they had completed all testing sessions up to that point. At the end of the study, participants and teachers at the schools attended a debriefing presentation given by the lead researcher (G.F.).

### Data reduction and analysis

On the Stop-Signal task, trials with errors and reaction-time (RT) outliers (defined as reaction times faster than 200 ms, slower than 2000 ms, and then if they were more than 3 SD above the participants' mean RT) were removed. Participants' Stop-Signal data were excluded from analysis if they had an outlying high rate of missing data (errors and outliers), determined by visual inspection of box-and-whisker plots.

To test if there was a latent factor for alcohol involvement, we performed a confirmatory factor analysis (CFA) using Mplus version 7.0 39 on three observed measures: frequency of drinking in the previous 6 months, frequency of getting drunk in the previous 6 months, and the API (for mean values, see Table 1). We checked for measurement invariance of the latent factor ‘alcohol involvement’ between the five time-points. All factor loadings were significant, ranging between 0.63 and 0.86. The overall fit of the model, assessed by the comparative fit index, was good [CFI = 0.97, root mean square error of approximation (RMSEA) = 0.05, χ^2^/d.f. = 1.85]. Full details are provided in Supporting information.

**Table 1:** Descriptive statistics; values are mean (standard deviation)

Session	1	2	3	4	5
Alc. cons freq.	1.01 (1.37)	1.29 (1.48)	1.45 (1.42)	1.72 (1.45)	1.80 (1.42)
‘Got drunk’ freq.	1.67 (4.96)	1.71 (4.61)	1.74 (3.93)	3.44 (7.80)	3.44 (8.26)
API	0.36 (0.92)	0.54 (1.24)	0.55 (1.09)	0.58 (1.07)	0.78 (1.23)
Disinhibition	273.23 (73.98)	268.67 (74.23)	259.59 (120.89)	277.08 (142.05)	274.75 (127.71)
Risk-taking	25.20 (10.70)	29.55 (11.80)	33.43 (11.96)	34.26 (12.03)	34.55 (11.02)
Delay discounting	0.51 (0.25)	0.52 (0.25)	0.50 (0.27)	0.49 (0.26)	0.48 (0.26)

Alc. cons. freq. = self-reported frequency of consuming alcohol in previous 6 months, coded 0 = never, 1 = a few times per year, 2 = once per month, 3 = once per fortnight, 4 = once per week, 5 = several times per week, 6 = almost every day. ‘Got drunk’ freq. = self-reported number of times drank to intoxication in previous 6 months. API = Alcohol Problems Index; values range from 0 (no problems) to 6 (most problems). Disinhibition = Stop-Signal reaction time (in milliseconds) from Stop Signal Task. Risk-taking = mean adjusted pumps from the BART. Delay discounting = area under the curve (AUC) values (reversed), derived from delay discounting task. For all impulsivity measures, higher values indicate higher impulsivity.

Delay discounting, disinhibition and risk-taking were not inter-related consistently at any time-point (see Supplementary Table S1). Indeed, when we performed an additional CFA in an attempt to create a latent impulsivity factor based on the three outcome measures from the impulsivity tasks (i.e. SSRT, BART-adjusted pumps and delay discounting AUC) this model did not converge, which suggests no latent factor of ‘impulsivity’.

Data were analysed using separate latent autoregressive cross-lagged models to examine prospective relationships between the alcohol involvement variable and each impulsivity measure in isolation. These models permit investigation of the relationships between variable *X* (e.g. delay discounting) at time 1 and variable *Y* (e.g. alcohol involvement) at time 2, after controlling for cross-sectional relationships between *X* and *Y* at both time-points, and for the stability paths of variables *X* and *Y* 40. Maximum likelihood robust (MLR) standard errors was used as the estimation method for all analyses. This method was chosen because the alcohol involvement measures were skewed, and MLR is robust to non-normality of data distributions 41.

To account for the clustering effect of school we considered multi-level modelling. However, in preliminary analyses we examined the intraclass correlations (ICC). ICCs for the majority of study variables were low (0.01–0.1) 42, indicating that any clustering effect of school was too small to have affected significantly the accuracy of SEs. Consequently, we proceeded with models that ignored any school effect.

## Results

### Study dropouts and missing data (see Supporting information)

Of the 287 participants who were enrolled initially into the study and completed the first session, 16 participants dropped out of the study across the 2-year period, resulting in a retention rate of 94.4%. There were no significant differences between study dropouts and study completers on any of the demographic or alcohol use measures, or on performance on any of the cognitive tasks at the first session (data not shown). An additional 18 participants missed at least one of the second, third or fourth sessions (of these, only one participant missed two sessions). We used full information maximum likelihood estimation (FIML, 43) to handle missing values for these participants.

### Descriptive statistics

In the first session, 64% of participants stated they had ever consumed an alcoholic drink. This percentage increased over subsequent sessions to 99% at the fifth and final session. Table 1 shows data from alcohol use and behavioural impulsivity tasks from all participants at each session: scores on the API, the frequency of alcohol consumption and the frequency of drinking to intoxication all increased over time. Risk-taking increased over the first three waves, but disinhibition and delay discounting showed no reliable changes over time.

### Cross-lagged models

**Figure 1:**
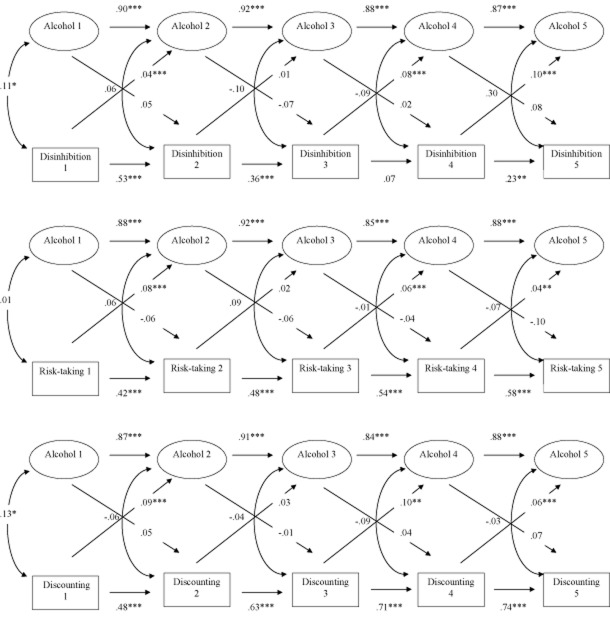
Cross-lagged models showing the reciprocal relationships between alcohol involvement (‘Alcohol’) and disinhibition as assessed with the Stop-Signal task (a), risk-taking as assessed with the Balloon Analogue Risk Task (b) and delay discounting (c). Values refer to standardized cross-loadings. *P < 0.05; **P < 0.01; ***P < 0.001

In the first model, we investigated the bidirectional relationships between disinhibition (inferred from SSRT), and alcohol involvement. See Fig. 1a. The overall model fit was acceptable [CFI = 0.93, RMSEA = 0.06, Tucker–Lewis Index (TLI) = 0.91, χ^2^/d.f. = 1.96, *P* < 0.01], and the stability paths between the five waves for the latent alcohol involvement factor were high, indicating that alcohol involvement across the five measurements was very stable. Stability paths for disinhibition were significant across all waves, apart from between waves 3 and 4 (there was a trend towards significance, *P* = 0.07). Cross-sectional relationships between alcohol involvement and disinhibition were significant only at waves 1 and 5. Most importantly, cross-lagged paths from disinhibition to alcohol involvement 6 months later were all significant, with the exception of disinhibition at wave 2 and alcohol involvement at wave 3, where the relationship fell short of significance (β = 0.01, *P* = 0.06). However, cross-lagged paths from alcohol involvement to disinhibition 6 months later were all non-significant. This suggests no prospective effects of alcohol involvement on disinhibition: it did not worsen in relation to alcohol involvement 6 months previously.

The analysis was repeated using risk-taking (based on the BART; Fig. 1b) and delay discounting (Fig. 1c) in separate models. Model fit for risk-taking was acceptable (CFI = 0.91, RMSEA = 0.07, TLI = 0.88, χ^2^/d.f. = 2.33, *P* < 0.01), although it was just below acceptable for delay discounting (CFI = 0.89, RMSEA = 0.08, TLI = 0.85, χ^2^/d.f. = 2.77, *P* < 0.01). The stability paths for alcohol involvement, as well as the stability paths for risk-taking and for delay discounting, were high in both models. For both risk-taking and delay discounting, the cross-lagged paths from the impulsivity measure predicting alcohol involvement 6 months later were significant: risk-taking and delay discounting assessed at waves 1, 3 and 4 predicted alcohol involvement 6 months later. Neither risk-taking nor delay discounting at wave 2 predicted alcohol involvement at wave 3, the same pattern that was seen for the Stop-Signal task. Importantly, the reverse paths (cross-lagged paths from alcohol involvement to risk-taking and delay discounting 6 months later) were all non-significant. As with the disinhibition data, we detected no prospective effects of alcohol involvement on risk-taking or delay discounting 6 months later: none of the behavioural impulsivity measures increased in relation to alcohol involvement 6 months previously.

## Discussion

Our cross-lagged models indicated that individual differences in performance on three behavioural impulsivity tasks each predicted a composite index of alcohol involvement 6 months later. These prospective relationships were consistent, as they were evident at three of the four 6-month intervals that we tested. However, we found no evidence for hypothesized alcohol-induced increases in behavioural impulsivity: individual differences in alcohol involvement did not predict subsequent impulsivity at any time-point.

As hypothesized, individual differences in disinhibition (as assessed with the Stop-Signal task) at the baseline assessment predicted alcohol involvement 6 months later. This finding is consistent with previous reports 14,15, and to our knowledge it is the first such demonstration of this relationship in an adolescent sample from outside North America. Equally important, our study is the first to demonstrate that delay discounting and risk-taking also predict alcohol involvement after fairly short follow-up periods of 6 months. Although previous studies have demonstrated cross-sectional associations between alcohol involvement and delay discounting in adolescents 12, no previous studies have investigated whether individual differences in delay discounting are associated with subsequent changes in alcohol consumption and problems, and so our study contributes important new data. Regarding risk-taking, a previous report 17 found that the rate of increase in risk-taking predicted a very small increase in the likelihood of alcohol involvement at subsequent assessment points [odds ratio (OR) = 1.02] in a sample who were slightly younger (9–12 years of age) than our own sample at the beginning of the study. In contrast, our results show that the absolute level of risk-taking predicted alcohol involvement only 6 months later, a relationship that was seen across multiple time-points.

We did not detect any evidence of changes in behavioural impulsivity as a consequence of heavy drinking during adolescence. Our study was the first to investigate this issue directly, and this is an important finding. However, it is possible that a longer follow-up period, or a focus on adolescents ‘at risk’ for development of substance use disorders 15 rather than a random sample as in the present study, may have yielded a different outcome. On a related note, while the frequency of drinking alcohol increased over time, the majority of our participants were drinking infrequently even at the end of the study. These are limitations of our study and an important avenue for future research, but we highlight the high financial costs associated with conducting longitudinal research over such long periods of time.

The model fit for our cross-lagged models was not exceptional (ideally, both CFI and TLI should be 0.95 or above, and RMSEA should be 0.05 or below). However, our fit indices can be described as acceptable 44. While our sample size was large, model fit may have been better with an even larger sample. In the delay discounting and risk-taking tasks, participants were responding for hypothetical rather than real financial rewards. We opted to use hypothetical rewards for ethical and practical reasons, and on the basis of previous studies that obtained comparable results from delay discounting tasks when real versus hypothetical rewards were used 45–47. In addition, other studies have shown that discounting rates for hypothetical monetary rewards are associated with alcohol use 12 and addictive behaviours more generally 48. Regarding the BART, although no previous studies have directly contrasted risk-taking behaviour when participants are responding for real versus hypothetical rewards, it is notable that risk-taking as measured by the BART is associated cross-sectionally with alcohol use regardless of whether hypothetical 8 or real 9 monetary rewards are used. However, one recent study suggests that the predictive validity of discounting tasks is superior when real rather than hypothetical rewards are used 49, and therefore it is important to replicate our findings using real financial rewards in the delay discounting and BART tasks.

Other limitations are our failure to record participant ethnicity, other drug use and trait (self-reported) impulsivity, so we were unable to evaluate the relative importance of behavioural measures of impulsivity after controlling for these other variables. Finally, we note some strengths of our study: the dropout rate was low, as was the percentage of missing data, thereby ensuring a high level of statistical power for all our primary analyses. No previous studies have tracked changes in performance on behavioural impulsivity tasks in relation to alcohol involvement over such an extended period of time, and we believe that the current study makes a very important contribution in this regard.

In summary, in the present study we explored longitudinal relationships between various indices of alcohol involvement and performance on behavioural impulsivity tasks. Across multiple time-points we found that disinhibition, delay discounting and risk-taking predicted alcohol involvement only 6 months later. Importantly, we found no evidence to suggest that heavy drinking had an impact on performance on any of these behavioural measures.

### Declaration of interests

None.
