# Supplementary material for: Multiple behavioural impulsivity tasks predict prospective alcohol involvement in adolescents
Source: Addiction. 2013 Aug 14;108(11):1916–23. doi: 10.1111/add.12283 (PMC4230409; doi:10.1111/add.12283)
Supplement: Supplementary file 1 — Appendix S1 Methods. [file add0108-1916-sd1.docx]

Supplementary Materials

Methods:

*Participant recruitment*: We initially contacted 105 schools to request their participation in the study. Of these, head teachers from nine schools expressed an interest in taking part, and we selected five of these schools for inclusion. We were successful in targeting schools that spanned the range of educational achievement (based on average exam results) and social deprivation (based on Government data), both at the initial recruitment stage and when selecting the final five schools for inclusion in the study. Each school was asked to provide a random selection of around 60 pupils, although the number actually recruited from each school ranged from 15 to 82. Four schools selected classes of pupils at random, and one school selected an entire academic year of pupils (i.e. multiple classes).

*Tasks administered*: In addition to the BART, stop-signal and delay-discounting tasks, participants completed two additional tasks: an alcohol-related visual probe task (based on the one described in ([1](#_ENREF_1))) and an alcohol-related Stimulus-Response Compatibility (SRC) task (based on the one described in ([2](#_ENREF_2))). These tasks measure attentional biases and automatic approach tendencies elicited by alcohol-related cues, respectively. We did not report results from these tasks because, unlike the impulsivity measures the internal reliability and between-session stability of these measures was poor. We briefly note that there were no consistent prospective relationships between performance on these measures and the latent factor for alcohol involvement, which is unsurprising given the poor reliability of the tasks. Details are available on request.

*Detailed description of tasks:*

#### Delay discounting task (DD ([3](#_ENREF_3))).

Participants made hypothetical choices between a relatively small sum of money that was available immediately versus a fixed larger sum which was available after a delay. We used the adjusting immediate amount procedure, as this is preferable to other versions of the task ([4](#_ENREF_4)). The value of the immediate reward and the length of the delay were adjusted on successive trials. The first trial presented the choice between £50 available immediately versus £100 available after a delay of one day. On subsequent trials the magnitude of the immediate reward was adjusted based on the decreasing adjustment algorithm described elsewhere ([3](#_ENREF_3)). In brief, if participants chose the immediate reward on a given trial, the magnitude of the immediate reward was decreased on the next trial, but if they had chosen the delayed reward, the magnitude of the immediate reward was increased on the subsequent trial. Participants made six choices at each of five delays (one day, one week, two weeks, one month, six months). Delay discounting was calculated using area under the curve (AUC; ([5](#_ENREF_5))). AUC values were inverted so that larger values indicated higher rates of delay discounting (i.e. high impulsivity). The internal reliability of the task was good: Cronbach’s α values (calculated from indifference points at the different delays) ranged between .84 and .87 (mean α = .86).

### Balloon Analogue Risk Task (BART ([6](#_ENREF_6))).

On each trial of the task, participants used the mouse to inflate an on-screen balloon. With each click, the balloon inflated and the amount of (hypothetical) money in a temporary bank increased by five pence (£0.05). Participants were instructed that at some point the balloon would burst; some balloons would reach the size of the screen while others would burst after only a few pumps and at this point all the money in the temporary bank would be lost. Participants could collect from the temporary bank at any point before the balloon burst by clicking on a button marked “Collect”. If they did so, money accumulated on that balloon was added to the safe bank, the balance of which was permanently displayed on-screen. Thus, each trial ended either when the balloon burst or when the participant moved their money to the safe bank. Participants were instructed to try to win as much hypothetical money as possible. The task comprised 30 trials, with a new balloon on each trial. On each trial, the balloon was randomly set to burst after between one and 128 pumps ([6](#_ENREF_6)). The dependent variable was the adjusted number of pumps, defined as the average number of pumps on trials when the participant banked their temporary funds (i.e., trials when the balloon did not burst). The internal reliability of the task was good: Cronbach’s α values (calculated from adjusted pumps in blocks of 10 trials) ranged between .77 and .85 (mean α = .82).

*Stop-Signal Task (*[*7*](#_ENREF_7)*)*

In this task, participants manually categorised visual ‘go’ stimuli as quickly as possible. On 25% of trials, an auditory ‘stop’ tone was presented; this tone signalled that participants should refrain from responding. On each trial, a white fixation cross (+) was presented in the centre of a black screen for 500ms before one of two white ‘go’ stimuli (an ‘X’ or a ‘O’) were presented until participants responded by pressing one of two keys on the keyboard, or until a 1000ms timeout had elapsed. On 25% of trials, the auditory ‘stop’ signal was presented through headphones. At the start of each block of trials, the stop signal delay (SSD), that is, the delay between onset of the ‘go’ stimulus and onset of the ‘stop’ stimulus was set at 250ms. On subsequent trials, SSD was altered in response to participants’ performance: if participants had successfully inhibited on the previous ‘stop’ trial, SSD on the next stop trial was increased by 50ms (making inhibition more difficult). If participants failed to inhibit on the previous trial, SSD was reduced by 50ms (making inhibition easier). There were six blocks of 32 trials, with eight stop-signal trials per block. The use of this ‘tracking procedure’ to manipulate SSD results in an SSD on which participants are able to inhibit on approximately 50% of trials, in which case Stop Signal Reaction Time (SSRT), can be computed as the average ‘Go’ reaction time minus the average SSD ([8](#_ENREF_8)). In some instances a reliable SSD could not be established; in these instances an alternative method was used to estimate SSRT ([9](#_ENREF_9)). High SSRTs indicate poor inhibitory control. The internal reliability of the task was good: Cronbach’s α values (calculated from SSRT in each block) ranged between .66 and .96 (mean α = .85).

*Counterbalancing of task order*: Participants completed the battery of five computerized tasks in one of five predetermined counterbalanced sequences. The within-session sequence remained the same across all of the testing sessions. Following the first wave of data collection we noted that some participants were very slow to complete the BART, although there was minimal variation in the time taken to complete the other tasks. As this presented an obstacle to successful data collection (all testing sessions had to be completed in under one hour in order to allow participants to get to their next lesson), we took the decision to modify the order of task presentation so that the BART was always completed last during waves 2-5 of data collection, so that it could be omitted if there was insufficient time. However, we always had sufficient time to administer all tasks, including the BART.

*Missing data*: Some data were missing from the BART (8 cases in session 1 and 1 case in session 3) and the delay discounting task (1 case in session 2, 5 cases in session 3, 1 case in session 4 and 2 cases in session 5) due to technical problems. Self-report data were missing from some sessions (<10% at each session). Considering all missing data (that is, data which was genuinely missing data plus data excluded due to excessive errors or outlying reaction times on the stop-signal task), no more than 10% of data were missing from any individual task or self-report measure at any one session. Despite missing 5-10% of data in sessions 2-4, less than 3.3% of data was missing from the fifth and final session. Little’s MCAR (missing completely at random) test was used to see if the data from each behavioural task were missing at random. Importantly, none of the alcohol use or behavioural task variables predicted missing data at the final session.

*Latent factor for alcohol involvement*: Maximum Likelihood Robust Standard Errors (MLR) was applied to control for the non-normal distribution of the self-report measures of alcohol consumption and problems. All factor loadings were significant ranging between .63 and .86 (see Supplementary Figure 1). Correlations between the measurement error of the latent indicators were defined (e.g. intoxication 1 with intoxication 2, intoxication 1 with intoxication 3 etc.). The overall fit of the model, assessed by the Comparative Fit Index, was good (CFI = .97, RMSEA = .05, χ^2^ / df = 1.85). Since the latent factor was assessed at five different time points, it was necessary to check for measurement invariance. We compared the constrained model, in which variances and factor loadings for alcohol involvement were constrained to be equal across the different time points, with an unconstrained model in which no constraints were imposed. The Bayesian Information Criteria (BIC) slightly favoured the unconstrained model (BIC_constrained = 30011 (86) BIC_unconstrained = 29995 (106)) indicating that measurement invariance was not ensured across all waves. Additional analysis revealed that intoxication frequency at wave 4 was the cause of measurement deviation of the latent factor. The BIC dropped down (from 29975 (94) to 29970 (88)) when the equality constraining of the factor loading of intoxication frequency at wave 4 was released. If we did not constrain the factor loading of intoxication frequency at wave 4, this increased the model fit (CFI = .93, RMSEA = .06 versus CFI = .90, RMSEA = .07 when constrained). However, since the overall model fit of the cross-lagged model was acceptable, at least weak measurement invariance was assumed ([10](#_ENREF_10)).

References

1. Field M, Mogg K, Zetteler J, Bradley BP. Attentional biases for alcohol cues in heavy and light social drinkers: The roles of initial orienting and maintained attention. Psychopharmacology. 2004;176(1):88-93.

2. Field M, Kiernan A, Eastwood B, Child R. Rapid approach responses to alcohol cues in heavy drinkers. Journal of Behavior Therapy and Experimental Psychiatry. 2008;39(3):209-18.

3. Du W, Green L, Myerson J. Cross-cultural comparisons of discounting delayed and probabilistic rewards. Psychological Record. 2002;52(4):479-92.

4. Holt DD, Green L, Myerson J. Estimating the subjective value of future rewards: Comparison of adjusting-amount and adjusting-delay procedures. Behavioural Processes. 2012;90(3):302-10.

5. Myerson J, Green L, Warusawitharana M. Area under the curve as a measure of discounting. Journal of the Experimental Analysis of Behavior. 2001;76(2):235-43.

6. Lejuez CW, Richards JB, Read JP, Kahler CW, Ramsey SE, Stuart GL, et al. Evaluation of a behavioral measure of risk taking: The balloon analogue risk task (BART). Journal of Experimental Psychology: Applied. 2002;8(2):75-84.

7. Bitsakou P, Psychogiou L, Thompson M, Sonuga-Barke EJS. Inhibitory deficits in attention-deficit/hyperactivity disorder are independent of basic processing efficiency and IQ. Journal of Neural Transmission. 2008;115(2):261-8.

8. Band GPH, van der Molen MW, Logan GD. Horse-race model simulations of the stop-signal procedure. Acta Psychologica. 2003;112(2):105-42.

9. Cohen JR, Asarnow RF, Sabb FW, Bilder RM, Bookheimer SY, Knowlton BJ, et al. Decoding developmental differences and individual variability in response inhibition through predictive analyses across individuals. Frontiers in Human Neuroscience. 2010;4.

10. Conroy DE, Metzler JN, Hofer SM. Factorial Invariance and Latent Mean Stability of Performance Failure Appraisals. Structural Equation Modeling. 2003;10(3):401-22.

Supplementary Table 1: Inter-correlations between behavioral measures at each time point. Values are Pearson’s correlations, two-tailed. * = p < .05, ** = p < .01

*First session*

Risk-taking Discounting

Disinhibition -.09, p = .14 .10, p = .11

Discounting .01, p = .86

*Second session*

Risk-taking Discounting

Disinhibition -.18, p = .00** .02, p = .70

Discounting .02, p = .71

*Third session*

Risk-taking Discounting

Disinhibition .12, p = .05 .03, p = .68

Discounting -.01, p = .88

*Fourth session*

Risk-taking Discounting

Disinhibition .01, p = .87 .07, p = .28

Discounting -.09, p = .13

*Fifth session*

Risk-taking Discounting

Disinhibition -.05, p = .46 -.03, p = .58

Discounting -.06, p = .32

Supplementary Figure 1: Latent factor for alcohol involvement at each time point.

Intox3

Often3

Intox2

Often2

.66

.86

.79

.76

.67

.79

.68

.70

.63

.79

.71

.79

.76

.76

.71

Intox1

API1

Often1

API3

API2

Often4

Intox4

API4

API5

Intox5

Often5

Factor loadings are all significant at p < .001

Often = how often alcohol consumed, intox = frequency of drinking to intoxication in last six months, API = Alcohol Problem Index
